# Histone Acetylation Enhancing Host Melanization in Response to Parasitism by an Endoparasitoid Wasp

**DOI:** 10.3390/insects15030161

**Published:** 2024-02-27

**Authors:** Kun Jiang, Yan Zhou, Wen Cui, Yan-Wei Han, Pei Chen, Gui-Ming Liao, You-Ming Hou, Bao-Zhen Tang

**Affiliations:** 1 State Key Laboratory of Ecological Pest Control for Fujian and Taiwan Crops, Fujian Agriculture and Forestry University, Fuzhou 350002, China; jiang__kun1998@163.com (K.J.); ymhou@fafu.edu.cn (Y.-M.H.); 2 Fujian Provincial Key Laboratory of Insect Ecology, Department of Plant Protection, Fujian Agriculture and Forestry University, Fuzhou 350002, China

**Keywords:** *Octodonta nipae*, *Tetrastichus brontispae*, parasitism, epigenetics, histone acetylation, melanization

## Abstract

**Simple Summary:**

Endoparasitic wasps are nature’s pest controllers, targeting various harmful insects. They lay their eggs inside these pests and release special substances (like viruses or venoms) that change the way the pest’s immune system works. However, we are still uncovering exactly how these changes happen. Our research looks into how certain wasps, which do not use these viruses, can still affect their host’s immune system. We studied this using a specific wasp–beetle pair as an example. We found that these wasps can control a crucial part of the beetle’s immune response through a process that modifies the beetle’s genetic material, specifically by adjusting certain markers on the genes that are important for immunity. This discovery sheds light on the sophisticated ways wasps can suppress the immune systems of their hosts, making them more effective at controlling pest populations.

**Abstract:**

Endoparasitoids are insects that develop within other insects, employing unique strategies to enhance their offspring’s survival. They inject polydnavirus and/or venom into their hosts along with eggs, effectively suppressing the host’s immune system. Polydnavirus from Braconidae and Ichneumonidae wasps can integrate into the host’s genome to express viral genes using the host’s transcription systems. However, the ability of parasitoids without polydnavirus to manipulate host gene expression remains unclear. Lysine acetylation (LysAc), a post-translational modification critical for gene regulation, is hypothesized to be used by endoparasitoids lacking polydnavirus. We utilized the Chalcidoidea wasp *Tetrastichus brontispae*, which lacks polydnavirus, as an idiobiont endoparasitoid model to test this hypothesis, with pupae of the nipa palm hispid beetle *Octodonta nipae* as the host. Parasitism by *T. brontispae* resulted in the reduced expression of histone deacetylase Rpd3 and elevated levels of LysAc modification at histones H3.3K9 and H3.3K14 through proteomics and LysAc modification omics. The knockdown of Rpd3 increased the expression level of OnPPAF1 and OnPPO involved in the phenoloxidase cascade, leading to melanization in the host body whereby it resembled a mummified parasitized pupa and ultimately causing pupa death. This study enhances our understanding of how endoparasitoids employ histone acetylation to regulate immunity-related genes, offering valuable insights into their survival strategies.

## 1. Introduction

The employment of Hymenoptera parasitoid wasps as biological control agents against insect pests underscores the critical ecological and practical importance of dissecting parasitoid–host interactions [1]. These endoparasitoids, which develop within their hosts, face the challenge of overcoming complex physiological and immunological defenses through either evasion or neutralization strategies [2,3,4]. The evolutionary arms race between endoparasitoids and their hosts has spurred the development of sophisticated adaptive strategies aimed at modifying the host’s immunity and influencing its development and physiology. Central to these strategies is the deployment of virulence factors, such as venoms [5], polydnaviruses [6,7], and teratocytes [8]. Particularly for those species lacking polydnavirus, venom emerges as a key mechanism for breaching host defenses [2].

Advances in omics technologies have revolutionized our understanding of changes in host gene expression following parasitization, revealing significant alterations in immunity and development. These breakthroughs, alongside targeted functional experiments [9], have emphasized the considerable impact of parasitoids on modulating specific immunity-related host genes. Despite these advancements, fully comprehending the mechanisms through which parasitoids regulate host gene expression remains an ongoing endeavor. Within this context, epigenetic regulation emerges as a pivotal evolutionary mechanism for adaptation to environmental challenges, extending beyond mere genetic mutation [10,11,12]. Recent research has highlighted the ability of parasitoids to alter host epigenetic landscapes, thereby influencing gene expression. Braconidae parasitoids, such as *Snellenius manila* and *Cotesia vestalis*, employ polydnavirus- or teratocyte-derived microRNAs (Cve-miR-281-3p from teratocyte, Cve-miR-novel22-5p from CvBv, Sm-miR-199b-5p, and Sm-miR-2989 from SmBV) to regulate genes crucial for host development [13,14], demonstrating the sophisticated mechanisms at play. This backdrop emphasizes the need to explore the capabilities of parasitoids, particularly those lacking polydnavirus or teratocytes, in influencing host gene expression through other epigenetic means.

The symbiotic origins of parasitoid venoms, and their potential evolution through lateral gene transfer (LGT) from ancient viral symbionts, offer a fascinating glimpse into the complex evolutionary history of these interactions [15,16]. This evolutionary perspective, suggesting deep-rooted symbiosis between parasitoids and their viral partners, enriches our understanding of the diverse strategies employed by parasitoids to manipulate their hosts. This is exemplified by the idiobiont ichneumon wasp *Pimpla turionellae*, whose venom delivery system significantly alters host epigenetic states, thereby affecting global DNA methylation, histone lysine acetylation ratios, and miRNA profiles—a testament to the intricate dynamics governing host gene transcriptional reprogramming [17].

Central to this study is lysine acetylation (LysAc), a key post-translational modification that responds to environmental cues and plays a vital role in reversible epigenetic regulation prior to transcription initiation [18,19]. The equilibrium maintained by histone acetyltransferases (HATs) and histone deacetylases (HDACs) in modulating transcription and gene expression underscores the complexity of epigenetic regulation [20,21,22,23,24,25]. Our investigation focuses on the pupal endoparasitoid wasp *Tetrastichus brontispae* Ferrière (Hymenoptera: Eulophidae), which, in the absence of polydnavirus, relies on maternal venom as its primary virulence factor [26,27]. *T. brontispae* targets Chrysomelidae (Coleoptera) pests, including *Octodonta nipae* and *Brontispa longissimi*, in palm cultivation [28,29]. Previous studies have validated the significant impact of *T. brontispae* parasitization on immunity-related genes in *O. nipae* pupae [30]. Utilizing the *O. nipae-T. brontispae system*, with the available genomic resource for *O. nipae* (unpublished data), we aim to explore how *T. brontispae* might influence host gene expression through histone acetylation modifications. This study hypothesizes that *T. brontispae* can manipulate the transcriptional reprogramming of immunity-related genes by altering host histone acetylation/deacetylation patterns, thereby achieving effective biological regulation.

## 2. Materials and Methods

### 2.1. Insect Rearing

*Octodonta nipae* were collected from Canary Date Palm (*Phoenix canariensis* Hortulanorum ex Chabaud) in Fuqing City, Fujian Province, China. *T. brontispae* were initially sourced from mummified *B. longissima* pupae provided by the Chinese Academy of Tropical Agricultural Sciences in 2008. These were then cultured in our laboratory using newly exuviated, one-day-old *O. nipae* pupae as hosts for consecutive generations. Both species were maintained under controlled conditions in an artificial climate incubator at 25 ± 1 °C and 75 ± 5% relative humidity, with a 12 h light:12 h dark photoperiod, as detailed in previous studies [28,31]. The diet of *O. nipae* consisted of fresh Canary Island date palm leaves (*Phoenix canariensis*). Concurrently, *T. brontispae* were propagated using one-day-old *O. nipae* pupae as hosts. Post-emergence, the adult wasps received 10% sucrose solution for nutrition within a plastic container. For the parasitism experiments, one-day-old adult female *T. brontispae*, chosen for their optimal parasitism rate, were utilized.

### 2.2. Analysis of LysAc Modification by Western Blot

Hemolymph and fat body samples were collected from thirty *O. nipae* pupae at intervals of 24, 48, and 72 h post-parasitization (PP), alongside tissues from an identical number of un-parasitized pupae (UP) serving as controls. Fat body samples underwent total protein extraction using a Protein Extraction Kit (BestBio Biotechnology Co. Ltd., Shanghai, China), whereas hemolymph samples were analyzed directly without prior protein extraction. Protein concentrations were determined using a BCA kit (Labgic Technology Co., Ltd., Beijing, China).

Approximately 15 μg of proteins from each sample underwent Western blot analysis. Proteins were separated via 12% SDS-PAGE and subsequently transferred onto a nitrocellulose filter membrane with Trans-Blot SD (Bio-Rad, Hercules, CA, USA). The membrane was then blocked using 5% skim milk for one hour at room temperature before being washed. Immunoblots were incubated with a pan anti-acetyllysine mouse monoclonal antibody (1:1000 dilution, PTM-101, PTM Biolabs Inc., Hangzhou, China) as the primary antibody, followed by horseradish peroxidase-conjugated goat anti-mouse IgG (1:1000 dilution, Thermo Fisher Scientific, Pleasanton, CA, USA) as the secondary antibody. Signals were detected using an Amersham™ ECL Select™ Western Blotting Detection Reagent (Global Life Sciences Solutions USA LLC, Marlborough, MA, USA) and visualized on an Amersham Imager 600 QC (GE Healthcare, Marlborough, MA, USA).

### 2.3. Analysis of LysAc Modification through the Combination of 4D Label-Free Proteomics and LysAc Modification Omics

Based on the Western blot results, proteins were extracted from 100 *O. nipae* pupae at 48 h post-parasitization and from an equivalent number of un-parasitized pupae for comparison. Protein extraction followed the same procedure as previously outlined. Equal amounts of protein from both the PP and UP groups underwent trypsinization, vacuum drying, and desalting procedures at PTM Biolabs Inc., Hangzhou, China.

For proteomic analysis, tryptic peptides were resuspended in solvent A (0.1% formic acid and 2% acetonitrile in water) and loaded onto a custom-made reversed-phase analytical column (25 cm length, 75/100 μm i.d.). Peptides were eluted using a gradient from 6% to 24% solvent B (0.1% formic acid in acetonitrile) over 70 min, increased from 24% to 35% in 14 min, and then, to 80% in 3 min, with a final hold at 80% for 3 min, at a constant flow rate of 450 nL/min using a NanoElute UHPLC system (Bruker Daltonics, Billerica, MA, USA). Following capillary source treatment, peptides were analyzed by a timsTOF Pro mass spectrometer (Bruker Daltonics), with the electrospray voltage set to 1.60 kV. Both precursor and fragment ions were detected across a broad *m*/*z* range of 100 to 1700 in MS/MS mode, using parallel accumulation serial fragmentation (PASEF) mode to select precursors with charge states from 0 to 5 for fragmentation. The system completed 10 PASEF-MS/MS scans per cycle, with dynamic exclusion set to 30 s.

For LysAc modification omics, affinity enrichment was conducted. Tryptic peptides in NETN buffer (100 mM NaCl, 1 mM EDTA, 50 mM Tris-HCl, 0.5% NP-40, pH 8.0) were incubated overnight at 4 °C with anti-acetyllysine antibody-conjugated agarose beads (PTM-104, PTM Biolabs Inc.) under gentle agitation. Peptides were eluted with 0.1% trifluoroacetic acid, vacuum-dried, and desalted using C18 ZipTips (Millipore, Burlington, MA, USA) before LC-MS/MS analysis, which proceeded as above.

Data were processed using the MaxQuant search engine (version 1.6.15.0), with tandem mass spectra matched against a comprehensive whole-length transcriptome database (including *Tetrastichus brontispae* and *Octodonta nipae*, totaling 62,796 entries) and a concatenated reverse decoy database. Trypsin/P was the designated cleavage enzyme, with up to two missed cleavages allowed. Precursor ion mass tolerance was set at 20 ppm for the initial search, narrowed to 5 ppm for the main search, and fragment ion mass tolerance at 0.02 Da. The fixed modification was carbamidomethyl, and variable modifications included N-terminal acetylation and Met oxidation. The false discovery rate (FDR) was maintained below 1%. Differential expression and LysAc modification analyses between the PP and UP groups utilized fold change (FC) criteria of >1.2 or <1/1.2 and a *p*-value threshold of <0.05, guiding comprehensive functional enrichment analyses.

### 2.4. RNA Isolation and Quantitative Real-Time PCR (qRT-PCR)

To investigate the influence of HATs and HDACs during parasitization, eight HAT genes and eight HDAC genes were targeted for qRT-PCR analyses at various intervals (within 48 h) following parasitization. RNA was extracted from both un-parasitized and parasitized pupae using Trizol reagents (Thermo Fisher Scientific) and subsequently converted to cDNA using the TransScript^®^ Uni All-in-One First-Strand cDNA Synthesis SuperMix for qRT-PCR (TransGen, Beijing, China). A minimum of three biological replicates were employed, with each replicate comprising ten pupae. Primer design was facilitated by the online tool Primer3 (V4.1.0) [32], with specifics provided in Supplementary Table S1. qRT-PCR was executed in triplicate per biological replicate on a 7500 Real-Time PCR System utilizing the SYBR Green protocol as previously outlined [33]. The *O. nipae ribosomal protein S3* (*rpS3*) gene was used as an internal control to adjust for potential variances in cDNA concentrations. Expression levels were determined with ABI 7500 system software (V2.0.6), ensuring the inclusion of the cited references.

### 2.5. Verification of Protein Expression and the LysAc Modification Level in Histone Proteins by Western Blot and qRT-PCR

For Western blot analysis, the protein samples used were the same as those in the omics analyses. Immunoblots were incubated with either anti-Acetyl-Histone H3 (Lys9) rabbit monoclonal antibody (PTM-112RM, PTM Biolabs) or anti-Acetyl-Histone H3 (Lys14) rabbit monoclonal antibody (PTM-113RM, PTM Biolabs) serving as the primary antibodies. The subsequent steps were conducted as previously described, maintaining consistency in the methodology.

For the qRT-PCR analysis, pupa samples were collected at several post-parasitization intervals: 6, 12, 24, 48, 72, and 96 h. *Histone H3* sequences were obtained from the comprehensive whole-length transcriptome database of *O. nipae*. The remaining experimental protocols were carried out in accordance with the methods outlined earlier, ensuring uniformity across different analyses.

### 2.6. RNA Interference

Based on the insights from qRT-PCR and proteomics analyses, *Rpd3*, a HDAC, was selected for RNA interference studies. Specific primers, conjugated to 20 bases of the T7 RNA polymerase promoter sequences and designed using E-RNAi [34], were utilized to amplify target DNA fragments of *Rpd3* and *eGFP* (details provided in Supplementary Table S1). Double-stranded RNA (dsRNA) synthesis was carried out using a HiScribe^®^ T7 Quick High Yield RNA Synthesis Kit (New England Biolabs, Ipswich, MA, USA), adhering to the manufacturer’s protocol. The produced dsRNA was quantified with a NanoDrop2000 spectrophotometer (Thermo Fisher Scientific) and its integrity verified by agarose gel electrophoresis. Subsequently, the dsRNA concentration was adjusted to 5000 ng/μL, with 200 nL injected per pupa using a Nanoliter2010 injector (World Precision Instruments, Sarasota, FL, USA). Injections of dsRNA targeting *eGFP* served as the negative control. Each experimental condition was replicated a minimum of three times, involving ten pupae per replicate. The efficacy of RNAi was assessed 24 h post-injection through qRT-PCR following the previously described methodology.

### 2.7. Phenotype Analysis 

Ten newly molted *O. nipae* pupae were chosen as a replicate for injections with either ds*Rpd3* or ds*eGFP*, ensuring three biological replicates per treatment to validate the results. Post-injection, the pupae were incubated under normal conditions. Observations for any phenotypic changes in the treated pupae were meticulously conducted and documented through daily photography up to the point of eclosion. In cases where mortality was observed before eclosion, the rate was accurately recorded, providing critical data for assessing the impact of the dsRNA treatments on pupal development and survival.

Based on observed phenotype changes, the expression levels of crucial genes within the phenoloxidase cascade, specifically *OnPPAF1* (*O. nipae* prophenoloxidase-activating factor 1) [35] and *OnPPO1* (*O. nipae* prophenoloxidase 1) [36], as well as several antimicrobial peptide genes, including *attacin*, *defensin*, and *lysozyme*, were analyzed 24 h post-ds*Rpd3* injection. The analysis was conducted according to the methodology described previously.

### 2.8. Capacity Assay of Clearance of Pathogen

Pupae were administered dsRNA treatments for 12 h, followed by a secondary injection of 100 nL *Escherichia coli* exhibiting green fluorescence (OD_600_ = 1) for an additional 12 h. From these treated pupae, approximately 3 μL of hemolymph was extracted from a cohort of five, and then, diluted to 30 μL with PBS. This diluted hemolymph sample was spread onto LB-ampicillin medium plates and incubated at 37 °C for 16 h. As a control, an injection of ds*eGFP* was employed. This experiment was replicated three times for each treatment to ensure reliability. Post-incubation, the culture plates were examined under a fluorescence microscope to identify and quantify fluorescent colonies, with their count and area meticulously documented.

### 2.9. Statistical Analysis

Data are presented as mean ± standard error. The analysis of qPCR data, pathogen clearance capacity, and triglyceride content across treatments was conducted utilizing Student’s *t*-test in GraphPad Prism version 8.3.0 for Windows (GraphPad Software, San Diego, CA, USA [https://www.graphpad.com, accessed on 29 October 2019]). A *p*-value of less than 0.05 was considered statistically significant.

## 3. Results

### 3.1. Initial Analysis of Regulation of Host Protein LysAc through the Parasitism of T. brontispae

**Figure 1:**
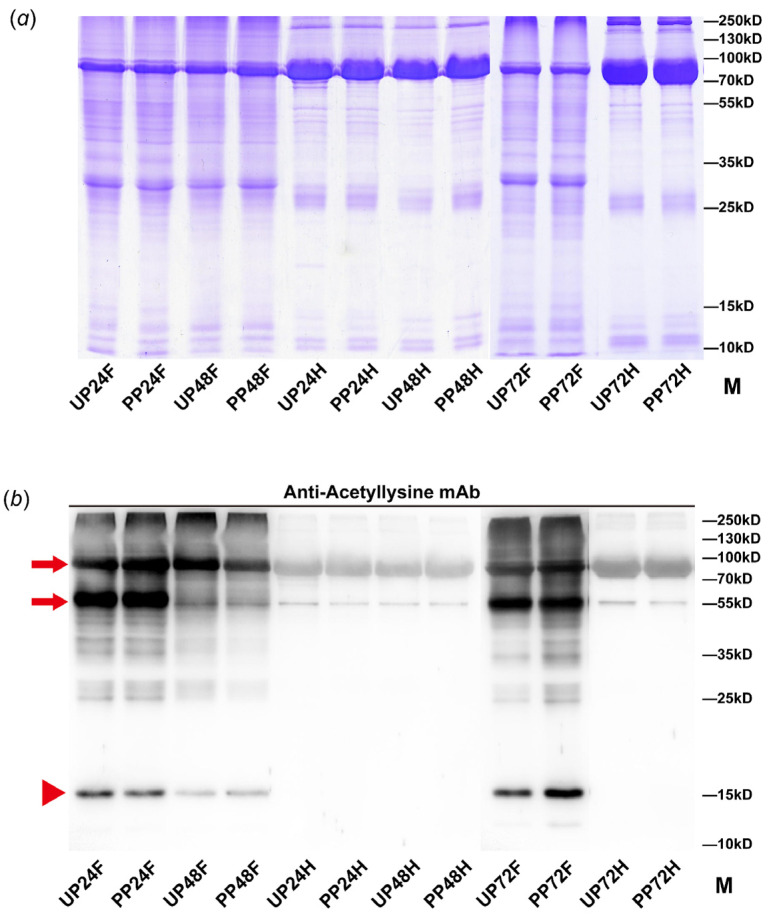
Analysis of LysAc modification level in tissues from post-parasitized (PP) and un-parasitized (UP) pupae using Coomassie brilliant blue gel staining (**a**) and Western blot (**b**). Hemolymph and fat body samples were collected at 24, 48, and 72 h post-parasitization. Tissues from un-parasitized pupae were collected simultaneously as controls. To ensure accuracy, the quality of an equal amount of protein in each lane was verified through Coomassie brilliant blue gel staining. The samples were then subjected to immunoblot analysis using a pan anti-acetyllysine mouse monoclonal antibody. The band with a molecular weight of approximately 15 kDa, indicative of histone protein, is highlighted with a red triangle, while bands with higher molecular weights correspond to non-histone proteins. Among these, two obvious non-histone bands, 55 and 85 kDa, are highlighted with red arrows. F, fat body samples; H, hemolymph samples.

Parasitoid wasps strategically target the hemolymph and fat body tissues of their hosts, which are essential for nurturing their offspring. To explore this dynamic, we harvested these tissues from pupae at intervals of 24, 48, and 72 h post-parasitization, alongside tissues from un-parasitized pupae as controls. Proteins extracted from these samples underwent Western blot analysis using a LysAc-specific pan antibody to evaluate the LysAc landscape. The analysis identified two prominent protein bands with molecular weights of 55 and 75 kDa, surpassing the molecular weight of histones, pinpointed at approximately 15 kDa based on our transcriptome data (Figure 1). This discovery underscores the occurrence of LysAc across both histone and non-histone proteins. Significantly, a marked decrease in LysAc modification levels was observed in the fat body tissues at 48 h post-parasitization, highlighting this time point as being crucial for differential LysAc modulation between the UP and PP groups (Figure 1). This critical finding directed our subsequent omics analyses towards the 48 h post-parasitization mark, with the aim of uncovering the intricate mechanisms through which *T. brontispae* parasitism influences host protein regulation.

### 3.2. Dynamic Changes in Proteome and LysAc Modifications in Host Pupae after Parasitization

**Figure 2:**
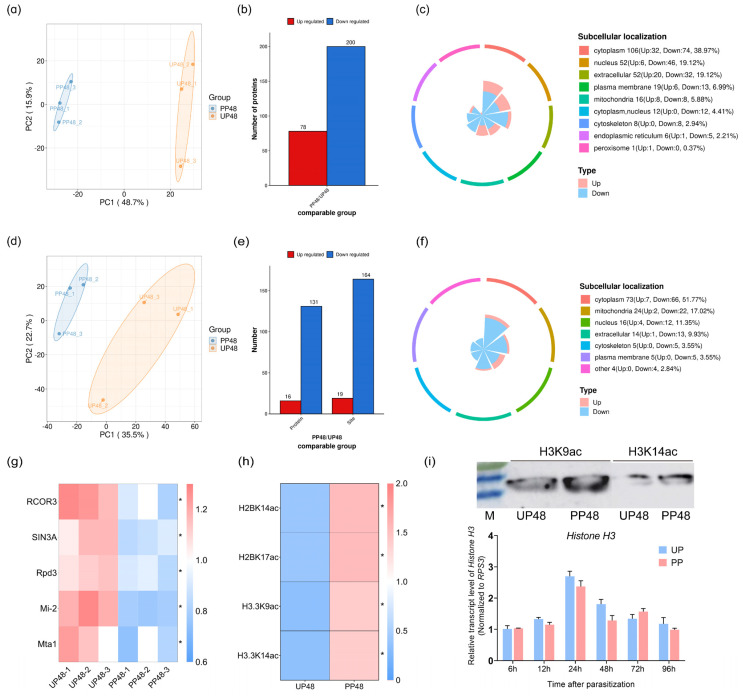
Dynamic changes in proteome and LysAc modifications in host pupae after parasitization. (**a**,**d**) PCA analyses of protein expression (**a**) and LysAc modifications (**d**) in two groups: PP48 (proteins from pupae at 48 h post-parasitization) versus UP48 (proteins from un-parasitized pupae at the same time point). (**b**,**e**) Summary of differentially expressed proteins and proteins with differential lysine acetylation modification between PP and UP, identified by a fold change (FC) > 1.2 or FC < 1/1.2 and *p*-value < 0.05. (**c**,**f**) Subcellular classification analysis of the differentially expressed proteins (**c**) and proteins with differential LysAc modification (**f**). (**g**) Heat map displaying proteins enriched in the GO term for protein deacetylase activity. (**h**) Heat map showing the level of LysAc modification in histone proteins. (**i**) Verification of the LysAc modification level of histone H3.3 via Western blot and qRT-PCR. Protein samples were identical to those used in omics analyses. Immunoblots were probed with the primary antibodies anti-Acetyl-Histone H3 (Lys9) rabbit monoclonal antibody and anti-Acetyl-Histone H3 (Lys14) rabbit monoclonal antibody. RNA samples were extracted at various time points post-parasitization: 6, 12, 24, 48, 72, and 96 h. The transcript level of histone H3 was normalized against the reference gene *ribosomal protein S3* (*rpS3*), with UP samples collected at 6 h serving as the baseline. “*” means significant difference at the 0.05 level between PP and UP.

To elucidate the regulatory effects of *T. brontispae* on LysAc modification, we utilized a comprehensive approach combining 4D-label-free proteomics and LysAc modification omics in *O. nipae* pupae at 48 h post-parasitization and un-parasitized controls. Principal component analysis (PCA) underscored a high correlation among biological replicates (Figure 2a,d), affirming the reliability of our dataset. The acetylome analysis unveiled 5515 unique LysAc sites across 1933 proteins (Supplementary Table S2). When normalized with proteomic data, 851 proteins with quantitative information were identified as acetylated, featuring 2660 unique LysAc sites (Supplementary Table S2). Applying the criteria of FC > 1.2 or FC < 1/1.2 and a *p*-value < 0.05 between the PP and NP groups, we identified 78 up-regulated and 200 down-regulated proteins (Figure 2b). To further our investigation, 19 unique sites on 16 proteins were found to be acetylated, whereas 164 sites on 131 proteins were deacetylated (Figure 2e). These differentially expressed and acetylated proteins predominantly resided in the cytoplasm, mitochondria, and extracellular regions (Figure 2c,f). Notably, proteins enriched in histone deacetylase activity and protein deacetylase activity were identified through Molecular Function GO enrichment analysis (Supplementary Table S3), with Rpd3, a HDAC, exhibiting decreased expression in the PP groups (Figure 2g).

KEGG pathway analysis further explored the potential functions of differentially acetylated proteins, revealing activation and enrichment in metabolism-related pathways (e.g., fructose and mannose metabolism, citrate cycle) and cell signaling pathways (e.g., cellular senescence, cGMP-PKG signaling pathway) (Supplementary Table S4). These findings highlight LysAc’s significant role in modulating host physiology following *T. brontispae* parasitization.

Special attention was given to histone proteins to compare their abundance and the LysAc modification levels between PP and NP. In line with our expectations, parasitization did not affect the abundance of these histone proteins (Supplementary Table S5). However, a notable increase in LysAc acetylation, including H2BK14ac, H2BK17ac, H3.3K9ac, and H3.3K14ac, was observed, with an approximate 4-fold increase in PP (Figure 2h and Table S5). Considering histone H3.3’s varied roles in transcription, genomic stability, and mitosis, Western blot analysis was conducted to confirm the increased acetylation of H3.3K9 and H3.3K14 (Figure 2i and Table S6), with qPCR validating that mRNA levels of histone H3 remained constant throughout the parasitization timeline (Figure 2i).

### 3.3. Expression Analysis of Host HATs and HDACs Induced by the Parasitism of T. brontispae

**Figure 3:**
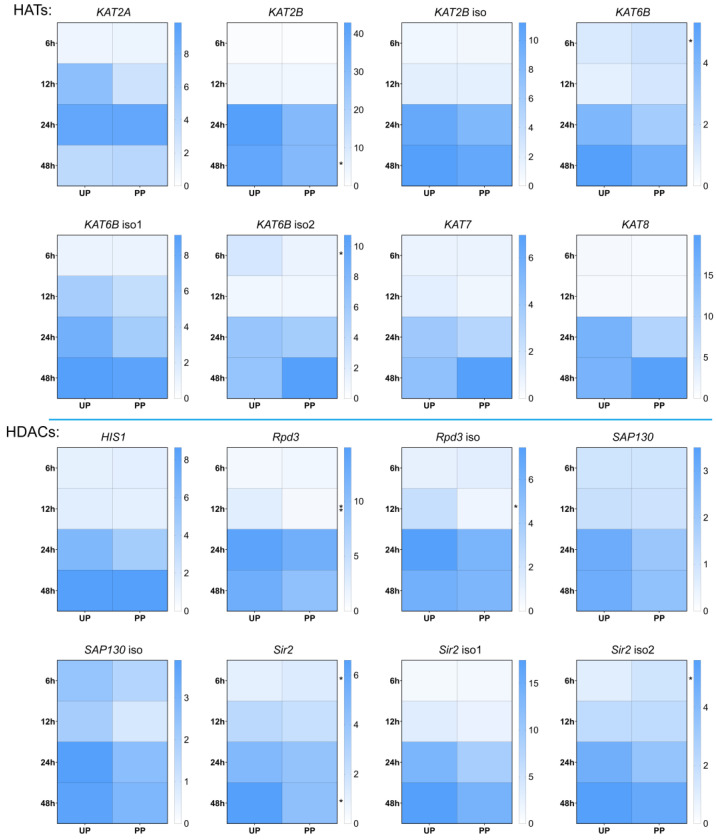
Transcript levels of HATs and HDACs. Eight HATs (*KAT2A*, *KAT2B*, *KAT6B*, *KAT7*, and *KAT8*) and eight HDACs (*HIS1*, *Rpd3*, *SAP130*, and *Sir2*) were selected for analysis. RNA samples were collected at various time points post-parasitization (6, 12, 24, and 48 h) (PP). Control RNA samples from un-parasitized pupae (UP) were collected simultaneously. Transcript levels were normalized to the reference gene *rpS3* and the UP sample collected at 6 h. * and ** indicate significant differences between PP and UP at the levels of *p* < 0.05 and *p* < 0.01, respectively.

Proteomic analysis unveiled a distinctive expression pattern of Rpd3 at 48 h post-parasitization compared to un-parasitized pupae, piquing our interest in *Rpd3*’s transcriptional dynamics during the early stages of parasitization. A pronounced decrease in *Rpd3* mRNA levels was observed as early as 12 h post-parasitization, showing a significant reduction of approximately 0.7-fold in both *Rpd3* and its isoforms (Figure 3). In addition to *Rpd3*, we assessed the mRNA expression levels of other *HDACs*—including *HIS*, *SAP130*, and *Sir2*—and HATs, namely *KAT2A*, *KAT2B*, *KAT6B*, *KAT7*, and *KAT8*.

Throughout the initial parasitization period, *Sir2* isoforms exhibited a decrease in mRNA levels by about 0.3-fold and 0.6-fold, respectively, while the transcripts of other *HDACs* remained largely unchanged (Figure 3). Moreover, a subtle yet significant variation in the abundance of *KAT2B* and *KAT6B* was detected between the PP and UP groups (Figure 3). This comprehensive analysis of *Rpd3*, along with other *HDACs* and *HATs*, during early parasitization highlights the nuanced regulatory mechanisms *T. brontispae* employs to modulate host epigenetic machinery, suggesting the targeted suppression of specific histone-modifying enzymes as a strategic adaptation to facilitate successful parasitism.

### 3.4. Role of Rpd3 in Prophenoloxidase Cascade

**Figure 4:**
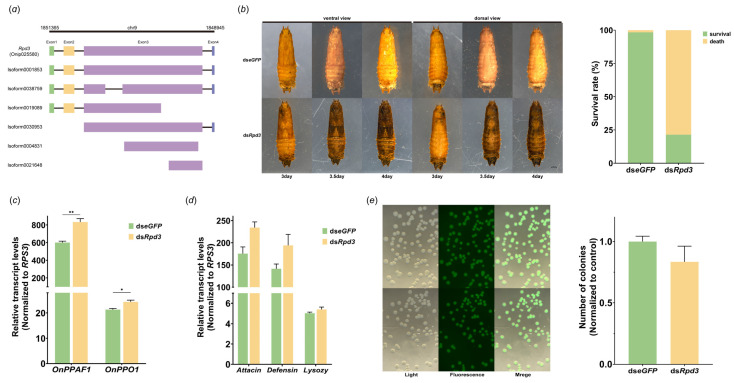
Involvement of Rpd3 in the regulation of prophenoloxidase cascade. (**a**) Analysis of alternative splicing of Rpd3. Chr9 represents chromosome 9 in the *O. nipae* genome, while 1,851,365 and 1,848,945 indicate the locations of Onip025580 (Rpd3) in the genome. Exon1–4 denote the four exons of this gene. (**b**) Phenotypic variation and survival analysis in *O. nipae* pupae following *Rpd3* knockdown. ds*eGFP* was used as a control. Observations were made at 3, 3.5, and 4 days post-injection of ds*Rpd3*. (**c**,**d**) Transcript levels of genes related to the prophenoloxidase cascade (**c**) and the antimicrobial peptide (**d**) 24 h post-*Rpd3* knockdown. * and ** represent significant differences between PP and UP at the levels of *p* < 0.05 and *p* < 0.01, respectively. (**e**) Evaluation of pathogen clearance in hemolymph after *Rpd3* knockdown. Pupae were treated with dsRNA for 12 h, and then, subjected to another injection of *E. coli* with green fluorescence for another 12 h. Cultured media were examined under a fluorescence microscope, counting and measuring the area of fluorescent colonies.

The parasitization by *T. brontispae* precipitated significant down-regulation in the expression of *Rpd3* at both the protein and transcript levels, leading us to further investigate its role in this biological process. Considering the presence of multiple isoforms of Rpd3, dsRNA was precisely engineered to target a conserved sequence within exon 3 (Figure 4a). Notably, the efficiency of RNA interference was observed to reach 60% at 24 h post-injection with ds*Rpd3*, demonstrating a substantial reduction in *Rpd3* expression (Figure S1). Fascinatingly, a marked phenotypic change was observed; the pupae’s heads began to darken at 72 h post-injection, with the discoloration extending to the thorax and eventually leading to the entire body turning black or resulting in death by 96 h post-injection (Figure 4b). This phenotype closely mimics that of mummified parasitized pupae, and calculations revealed the mortality rate to be approximately 80% (Figure 4b).

To elucidate the underlying cause of this dramatic transformation, we analyzed the expression levels of crucial genes within the phenoloxidase cascade, specifically *OnPPAF1* and *OnPPO1*, at 24 h after ds*Rpd3* injection. The results showed a statistically significant increase in the transcripts of both *OnPPAF1* and *OnPPO1*, albeit modest (OnPPAF1, *t*_4_ = 5.702, *p* = 0.0047; OnPPO1, *t*_4_ = 3.756, *p* = 0.0199) (Figure 4c).

Moreover, given previous reports of clip-domain serine proteases being involved in the synthesis of antimicrobial peptides, we assessed the expression of antimicrobial peptide genes and the hemolymph’s capacity to clear foreign pathogens following *Rpd3* knockdown. However, we observed no significant changes in the mRNA levels of these antimicrobial peptide genes or in the hemolymph’s pathogen clearance ability at 24 h following *Rpd3* interference (Attacin, *t*_4_ = 2.733, *p* = 0.0523; Defensin, *t*_4_ = 1.957, *p* = 0.1220; Lysozy, *t*_4_ = 1.496, *p* = 0.2089) (Figure 4d,e).

## 4. Discussion

Endoparasitoids have evolved an intricate repertoire of strategies to modulate host physiological pathways, creating an optimal environment for their offspring’s development [37]. Central to these strategies are virulence factors such as venoms and polydnaviruses, which are adept at altering host physiological responses to favor parasitoid development [38,39]. The integration of polydnaviruses into the host genome, a phenomenon observed in Braconidae and Ichneumonidae wasps, exemplifies a profound level of genomic integration and expression, underscoring a deep symbiotic relationship [40,41,42]. This context provides a unique lens through which the evolutionary origins of parasitoid venoms, possibly resulting from LGT from ancient viral symbionts, can be explored [15,16]. Such a perspective adds depth to our understanding of the co-evolutionary dynamics between parasitoids and their hosts, highlighting the complexity of these biological interactions.

Our study delves into the realm of epigenetic modifications, focusing on histone acetylation and deacetylation, to understand how *T. brontispae* influences host gene expression. The bracovirus of *C. plutellae* offers a compelling case of host manipulation through epigenetic modifications, introducing a variant histone H4 with an extended N-terminal tail into the host nucleosome, facilitating transcriptional reprogramming within the host [43,44]. This example underscores the pivotal role of histone modifications in the intricate relationship between parasitoids and their hosts, setting the stage for our investigation into *T. brontispae*’s epigenetic influence.

Initial analyses using anti-LysAc antibody-based Western blots revealed that the impact of lysine acetylation extends beyond histones to non-histone proteins, affecting vital metabolic and immune pathways post-parasitization. The observed predominance of lysine deacetylation, aligning with known transcription repression mechanisms [45,46], suggests a sophisticated strategy employed by *T. brontispae* to suppress host immunity. 

Our further comparative LysAc proteomics identified an increase in the acetylation levels of the histone proteins H3.3K9 and H3.3K14 in parasitized pupae, highlighting the dynamic interplay between HATs and HDACs in mediating these modifications [23,47]. The down-regulation of Rpd3, a class I HDAC previously identified in *Saccharomyces cerevisiae* as being involved in the acetylation modification of histone H4K12 [48] and correlated with the acetylation levels of histones H3 and H4 across several biological pathways in *Drosophila* and *Tribolium castaneum* [49,50], presents a sophisticated epigenetic regulatory mechanism employed by *T. brontispae*. This mechanism is further illustrated by the significant differences in the mRNA expression levels of *Sir2*, *KAT2B*, and *KAT6B* between parasitized and un-parasitized groups, emphasizing the nuanced role of Rpd3 and other HDACs and HATs in host–parasitoid interactions.

The physiological roles of Rpd3, from influencing lifespan and memory capacity in *Drosophila* to developmental stagnation in *T. castaneum* upon interference [51,52], further contextualize our findings. The knockdown of *Rpd3* in *O. nipae* pupae, resulting in black clumps and subsequent death, resembles mummified parasitized pupae and suggests a link to melanization processes [53]. This observation led us to investigate *OnPPAF1* and *OnPPO1* expression [35,36], pivotal in the phenoloxidase cascade, which, despite not showing significant fold changes post-Rpd3 silencing, aligns with enhanced host PO activity observed following parasitization [54].

Despite these significant findings, the absence of notable changes in antimicrobial peptide gene expression or pathogen clearance ability following *Rpd3* silencing, coupled with the modest fold changes in the expression of other *HDACs* and *HATs*, as well as *OnPPAF1* and *OnPPO1*, underscores the complexity of the interactions at play. This complexity suggests that the timeframe of our investigation may not have been sufficient to capture the extensive range of epigenetic and transcriptional shifts induced by *T. brontispae* parasitization. This observation underscores the necessity for further research to elucidate the temporal dynamics of these regulatory mechanisms. Additionally, future studies should focus on the role of venom as a factor in driving these epigenetic modifications, which could provide deeper insights into the mechanisms by which *T. brontispae* influences host gene expression.

In summary, this investigation sheds light on a detailed regulatory mechanism by which *T. brontispae* modulates host prophenoloxidase activity through Rpd3-mediated histone acetylation, emphasizing the critical role of histone acetylation in regulating prophenoloxidase expression. Our findings not only contribute to the understanding of the epigenetic regulatory strategies employed by idiobiont endoparasitoids lacking polydnaviruses and teratocytes, but also highlight the significance of histone acetylation in the regulation of host immune responses, offering new insights into the survival strategies of parasitoid offspring.

## Data Availability

The data that support the findings of this study are available from the corresponding author upon reasonable request.

## Supplementary Materials

The following supporting information can be downloaded at: https://www.mdpi.com/article/10.3390/insects15030161/s1, Figure S1: Interference efficiency of Rpd3; Table S1: Primers for qRT-PCR and RNAi; Table S2: Summary of MS/MS spectrum database search analysis; Table S3: Go enrichment analysis of differentially expressed proteins; Table S4: KEGG pathway enrichment analysis of differentially acetylated proteins; Table S5: Summary of histone lysine acetylation modification. Table S6: Analysis of Western blot result through calculation of integrated density; Original Western blot image of Figure S1: Figure S1—24 and 48 h samples, Figure S1—72 h samples; Original Western blot image of Figure S2i.
