# Supplementary material for: Histone Acetylation Enhancing Host Melanization in Response to Parasitism by an Endoparasitoid Wasp
Source: Insects. 2024 Feb 27;15(3):161. doi: 10.3390/insects15030161 (PMC10971516; doi:10.3390/insects15030161)
Supplement: Supplementary file 1 [file insects-15-00161-s001.zip › insects-2853087-supplementary/Figure 2i (original western blot).pdf]

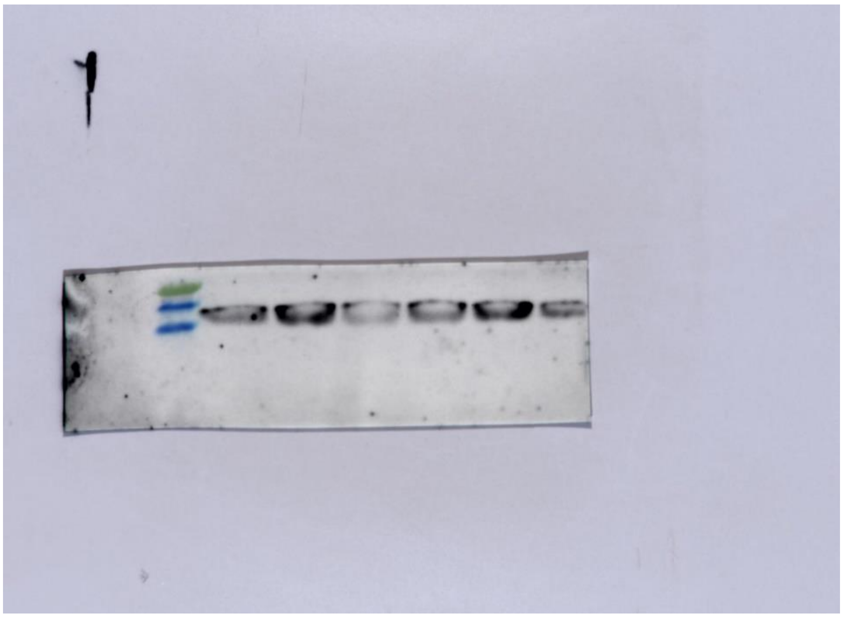

Antibody: H3K9ac (lane 1, Marker; lane 2-UP48; lane 3-PP48)

Lane 4, 5, 6 and 7 are other samples.

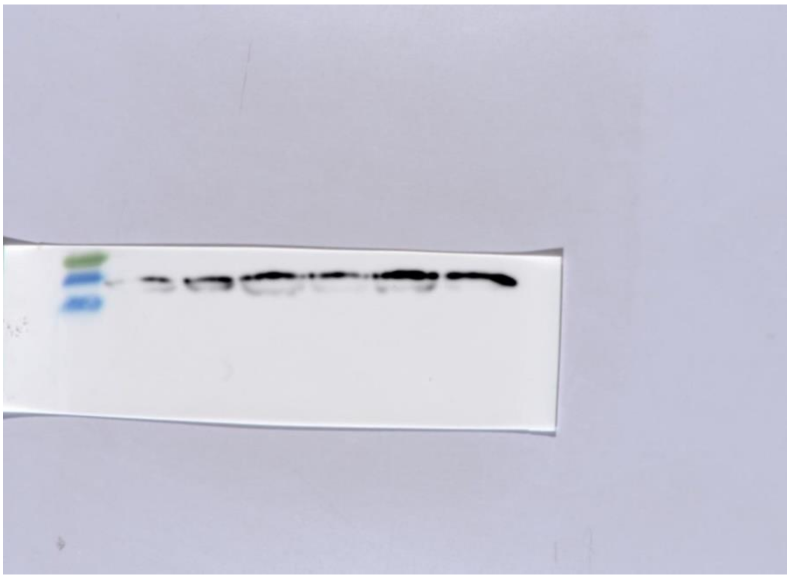

Antibody: H3K14ac (lane 1, Marker; lane 2-UP48; lane 3-PP48)

Lane 4, 5, 6 and 7 are other samples.
