# Supplementary material for: Histone Acetylation Enhancing Host Melanization in Response to Parasitism by an Endoparasitoid Wasp
Source: Insects. 2024 Feb 27;15(3):161. doi: 10.3390/insects15030161 (PMC10971516; doi:10.3390/insects15030161)
Supplement: Supplementary file 1 [file insects-15-00161-s001.zip › insects-2853087-supplementary/Figure1--24 and 48 h samples (original western blot).pdf]

Coomassie brilliant blue staining (15 ug protein/lane)

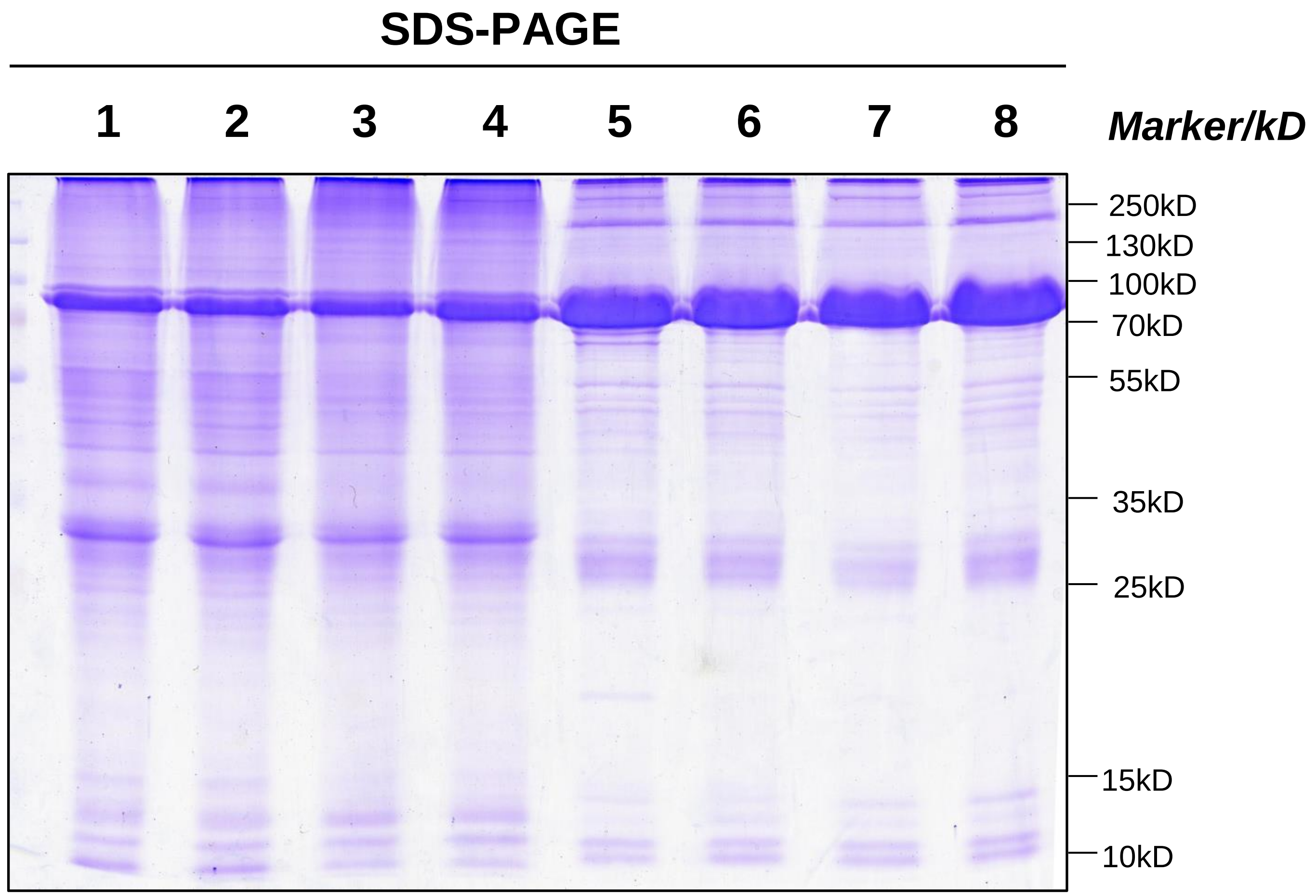

1: UP24F
2: PP24F
3: UP48F
4: PP48F
5: UP24H
6: PP24H
7: UP48H
8: PP48H

Western blotting with pan anti-acetyllysine antibody

WB: anti-acetyllysine

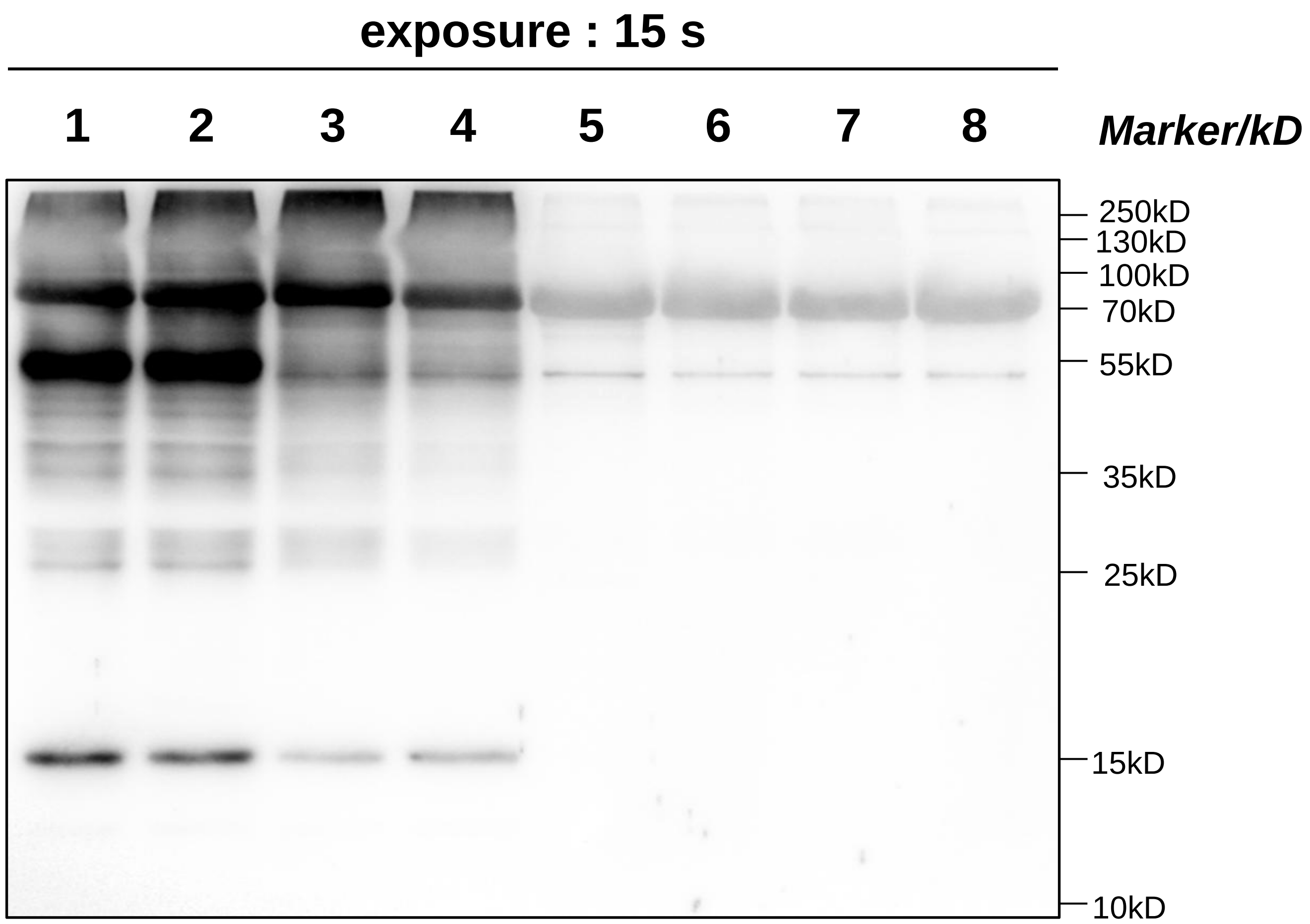

- 1: UP24F
- 2: PP24F
- 3: UP48F
- 4: PP48F
- 5: UP24H
- 6: PP24H
- 7: UP48H
- 8: PP48H

Western blotting with pan anti-acetyllysine antibody

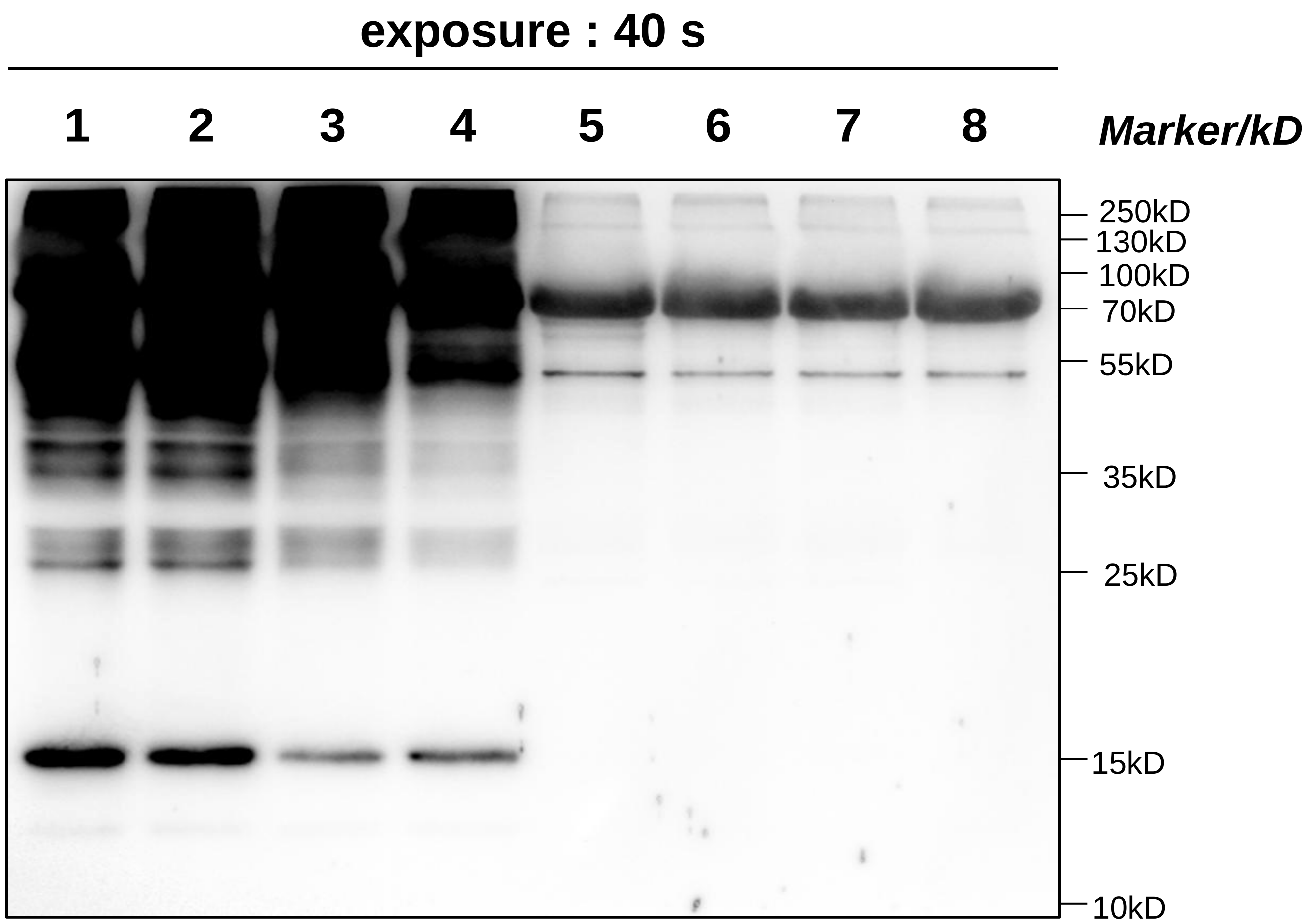

WB: anti-acetyllysine

1: UP24F
2: PP24F
3: UP48F
4: PP48F
5: UP24H
6: PP24H
7: UP48H
8: PP48H

1. 15 μg protein / lane
2. Primary antibody : Anti-acetyllysine Antibody（PTM-101; Lot: 12838533L303; 1:1000 dilution)
3. 2nd antibody: Thermo, Pierce, Goat anti-Mouse IgG, (H+L), Peroxidase Conjugated, 31430, 1:10000 dilution

Western blotting with pan anti-acetyllysine antibody

WB: anti-acetyllysine

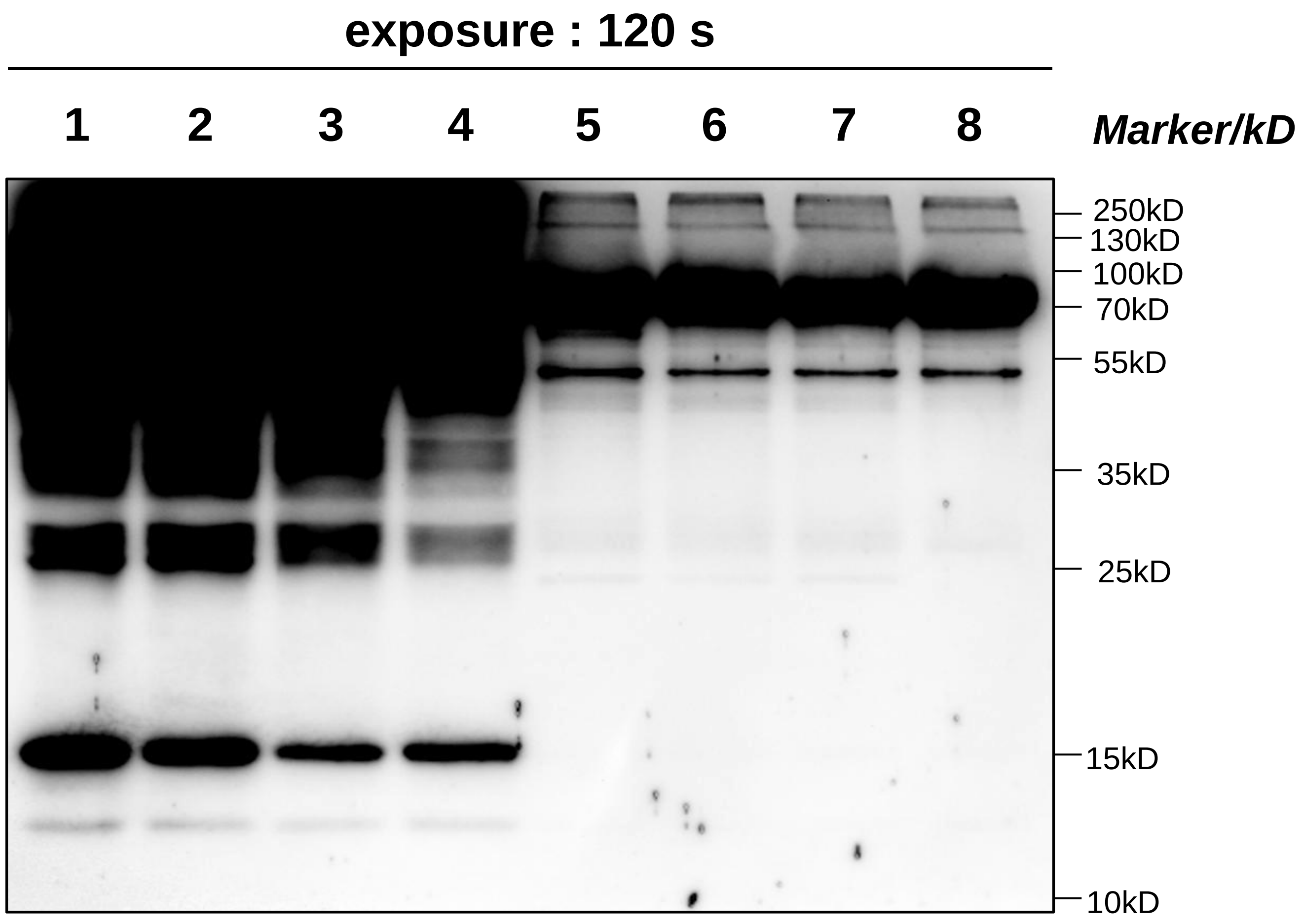

1: UP24F
2: PP24F
3: UP48F
4: PP48F
5: UP24H
6: PP24H
7: UP48H
8: PP48H
