# Supplementary material for: Histone Acetylation Enhancing Host Melanization in Response to Parasitism by an Endoparasitoid Wasp
Source: Insects. 2024 Feb 27;15(3):161. doi: 10.3390/insects15030161 (PMC10971516; doi:10.3390/insects15030161)
Supplement: Supplementary file 1 [file insects-15-00161-s001.zip › insects-2853087-supplementary/Figure1--72 h samples (original western blot).pdf]

Coomassie brilliant blue staining : 15 ug protein/lane (lane 3, 4, 7 and 8 are other samples)

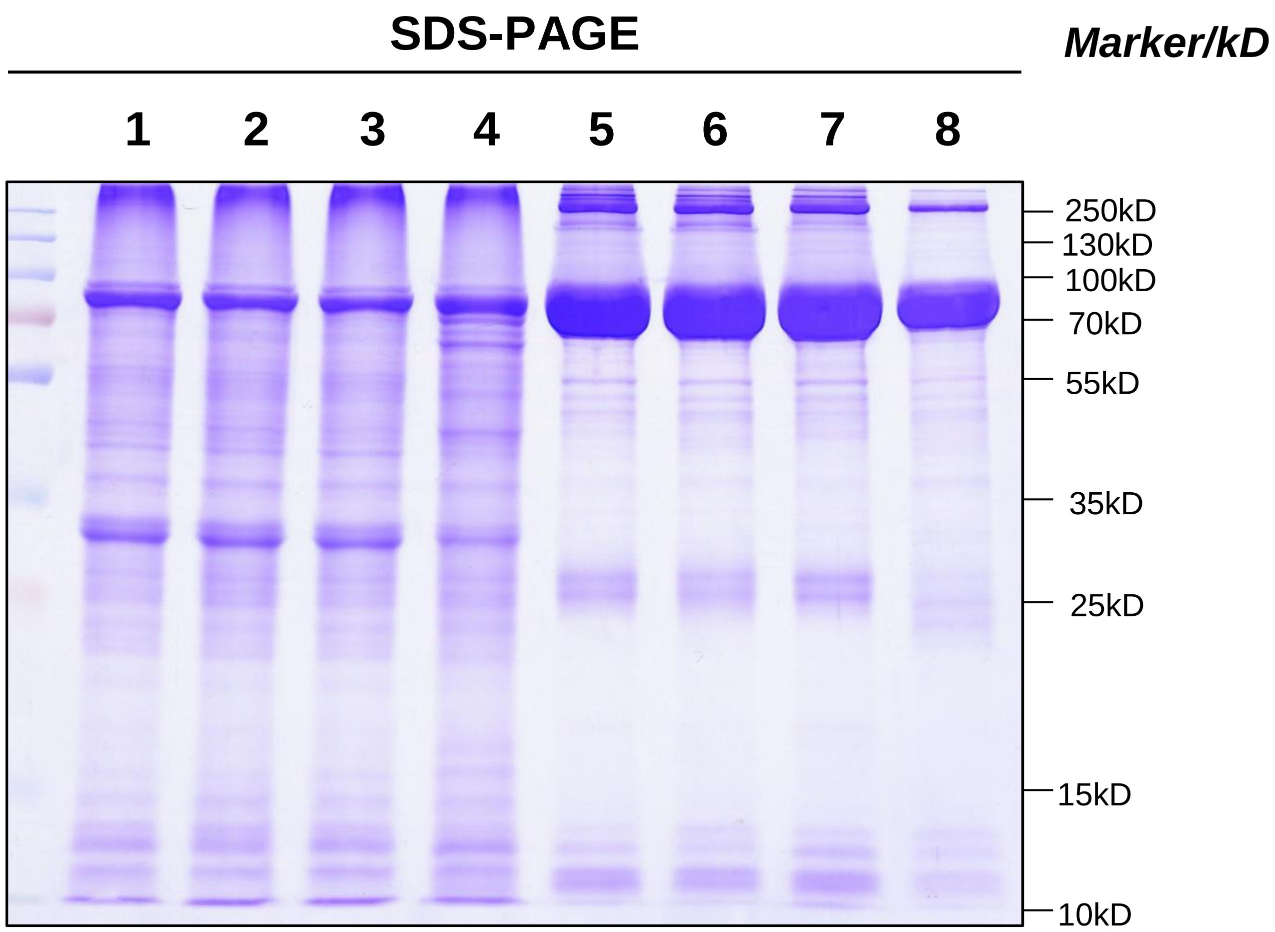

1: UP72F
2: PP72F
5: UP72H
6: PP72H

Western blotting with pan anti-acetyllysine antibody

WB: anti-acetyllysine

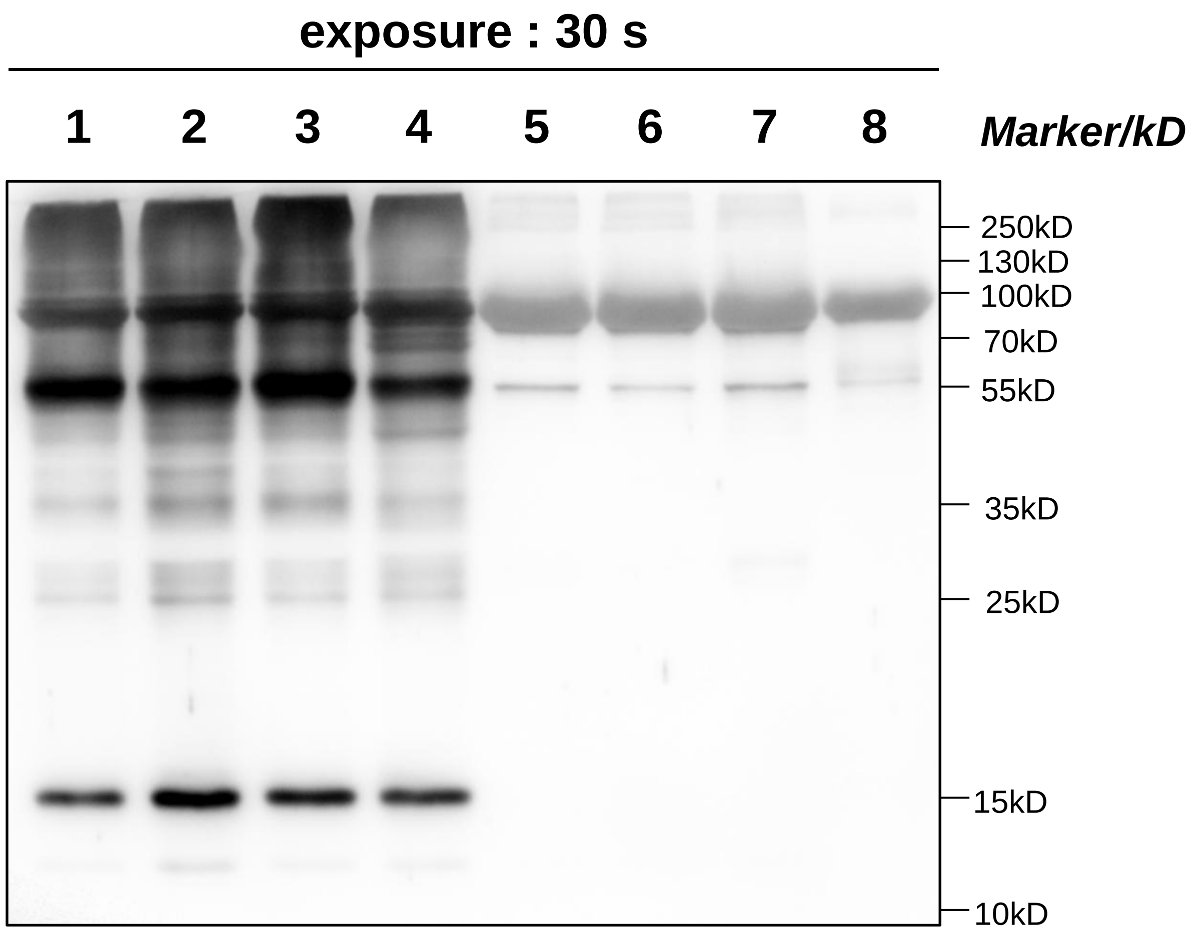

1: UP72F
2: PP72F
5: UP72H
6: PP72H

Western blotting with pan anti-acetyllysine antibody

WB: anti-acetyllysine

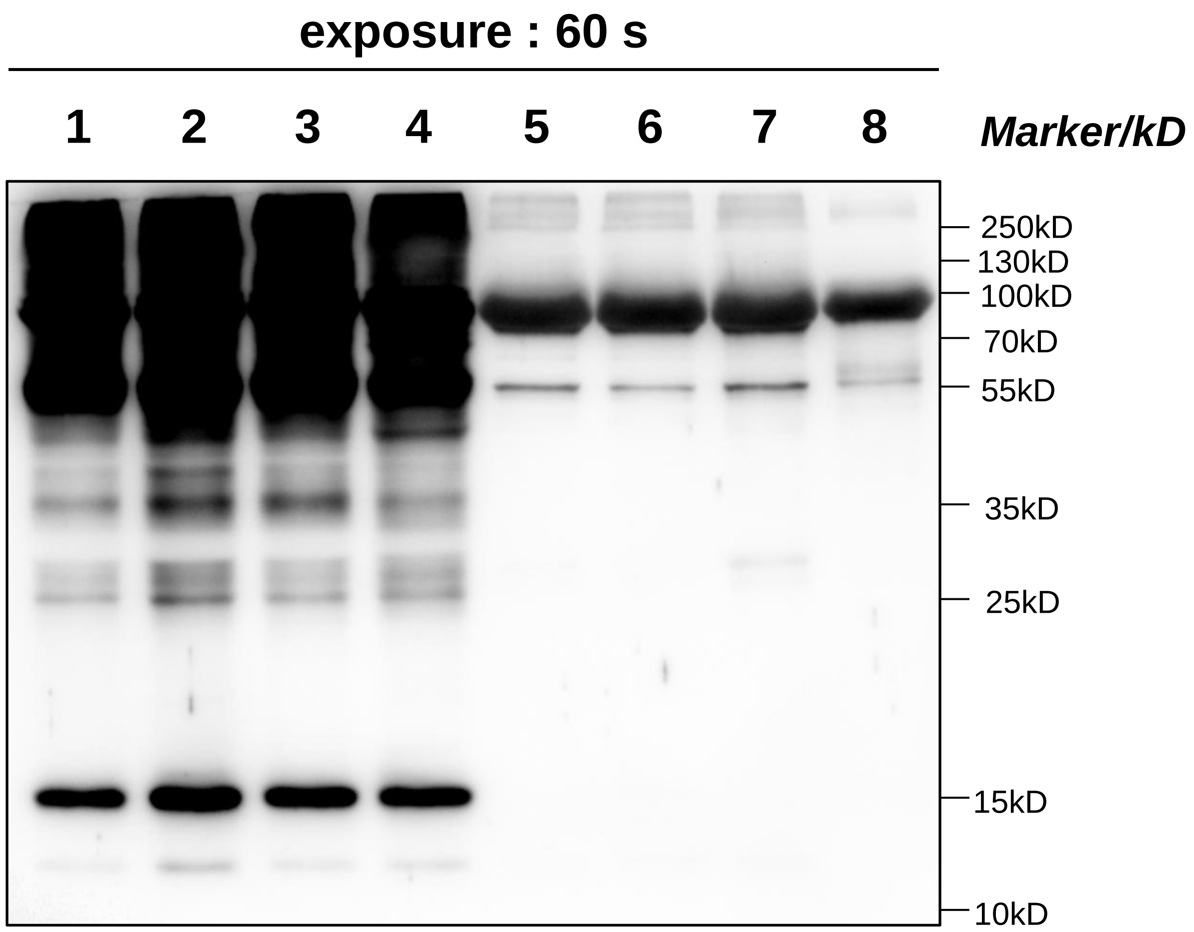

1: UP72F
2: PP72F
5: UP72H
6: PP72H

Western blotting with pan anti-acetyllysine antibody

WB: anti-acetyllysine

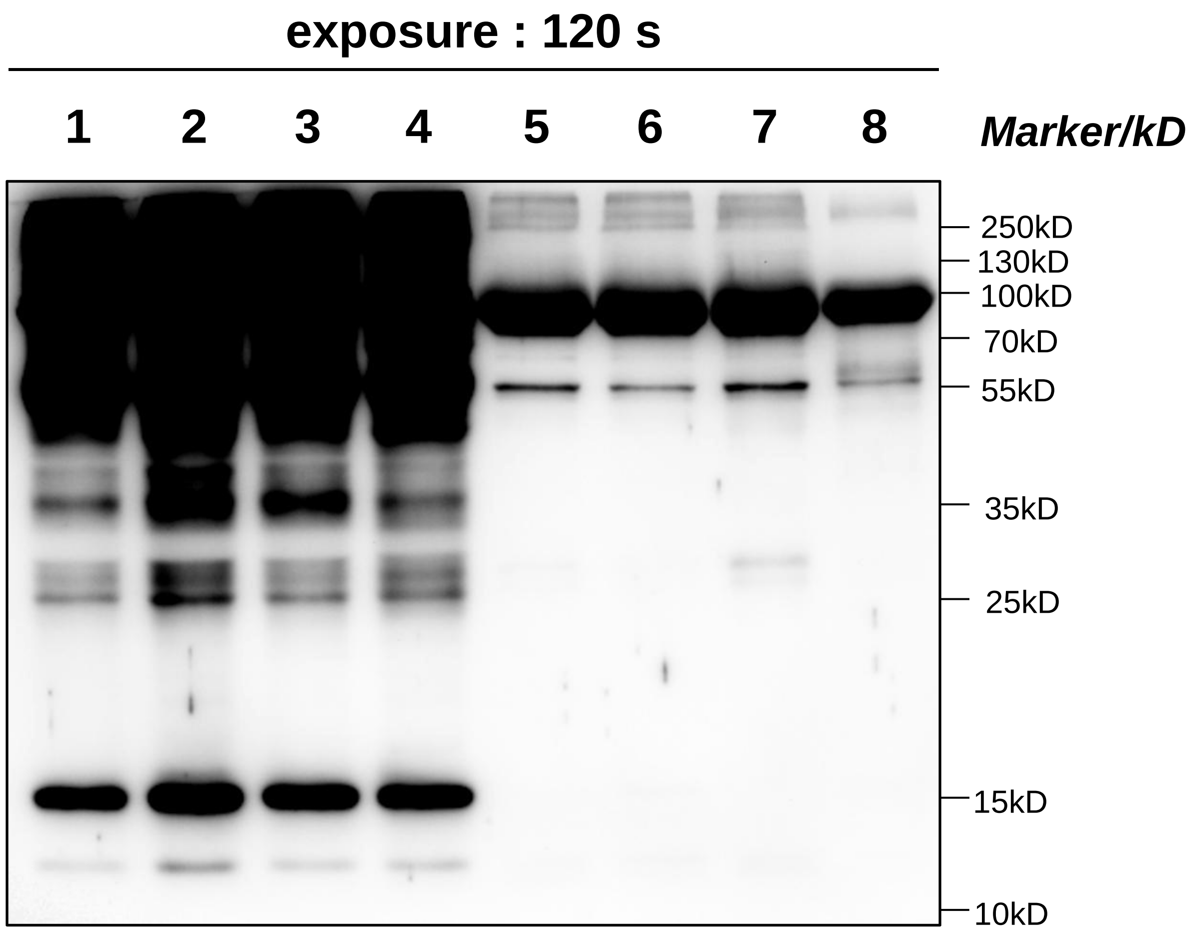

1: UP72F
2: PP72F
3: UP96F
4: PP96F
5: UP72H
6: PP72H
7: UP96H
8: PP96H
