# Supplementary figures and images for: Histone Acetylation Enhancing Host Melanization in Response to Parasitism by an Endoparasitoid Wasp
Source: Insects. 2024 Feb 27;15(3):161. doi: 10.3390/insects15030161 (PMC10971516; doi:10.3390/insects15030161)

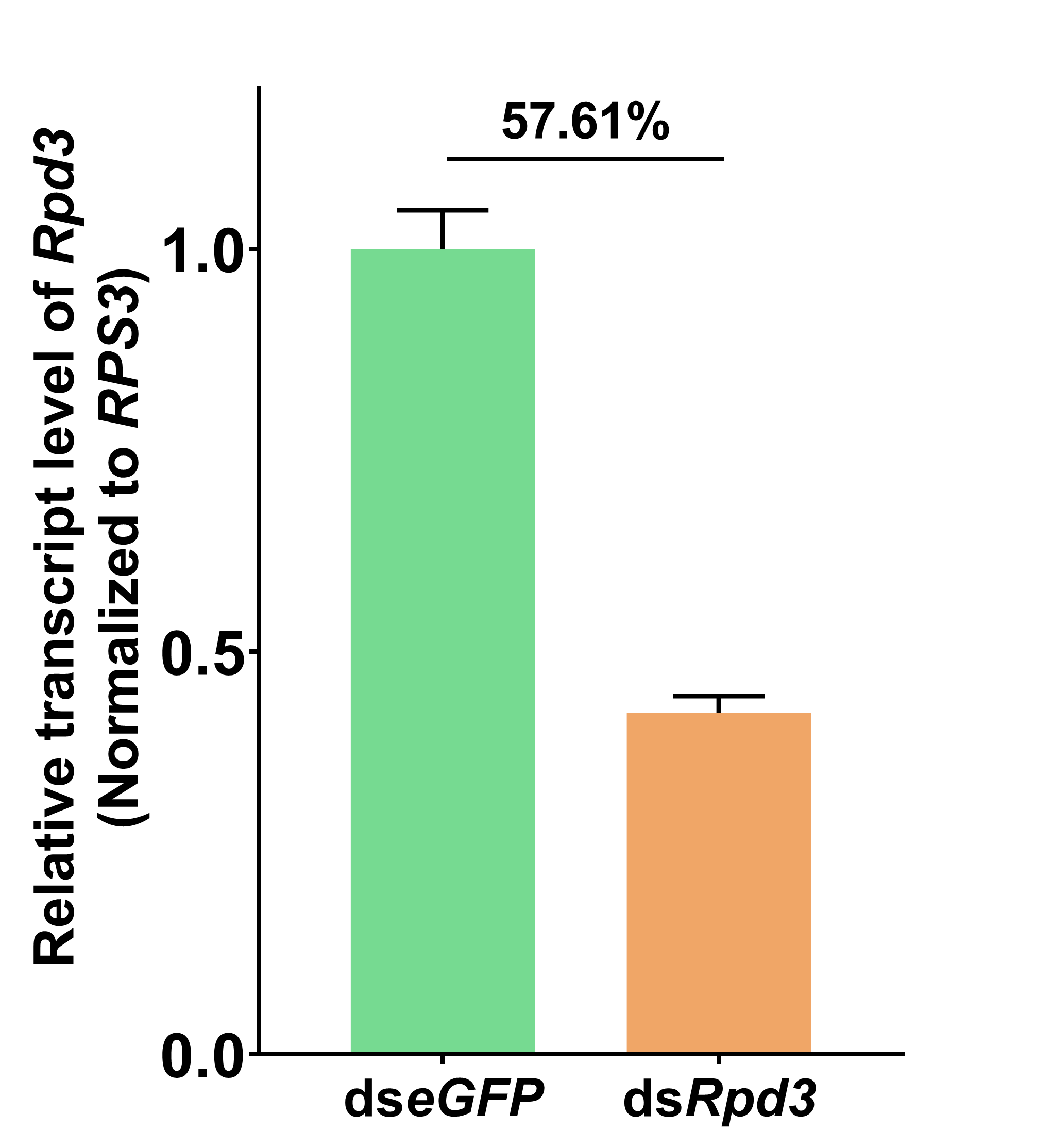

Supplement: Supplementary file 1 [file insects-15-00161-s001.zip › insects-2853087-supplementary/Figure S1 Interference efficiency of Rpd3.tif]
